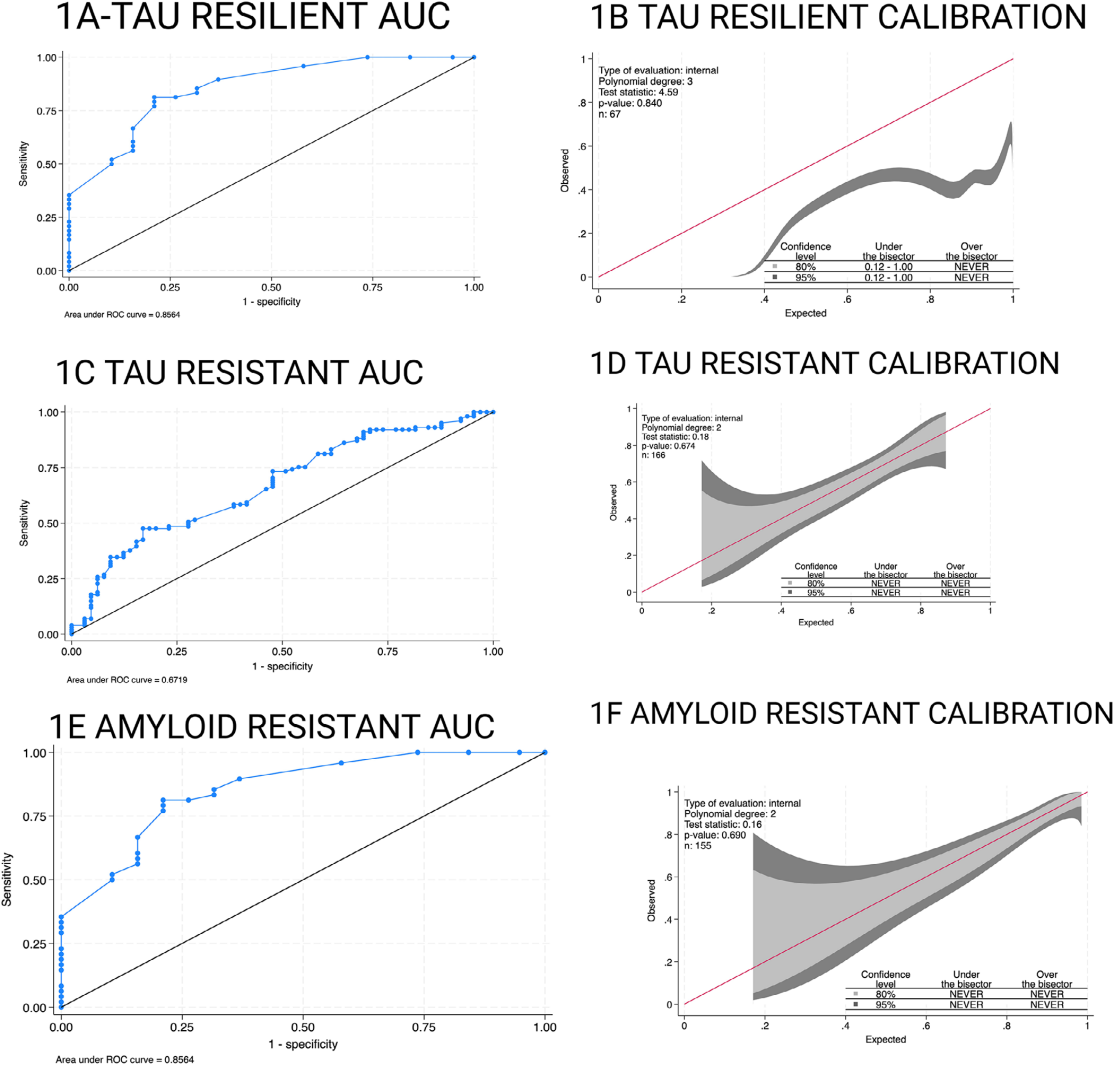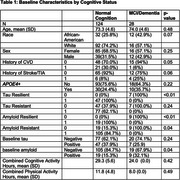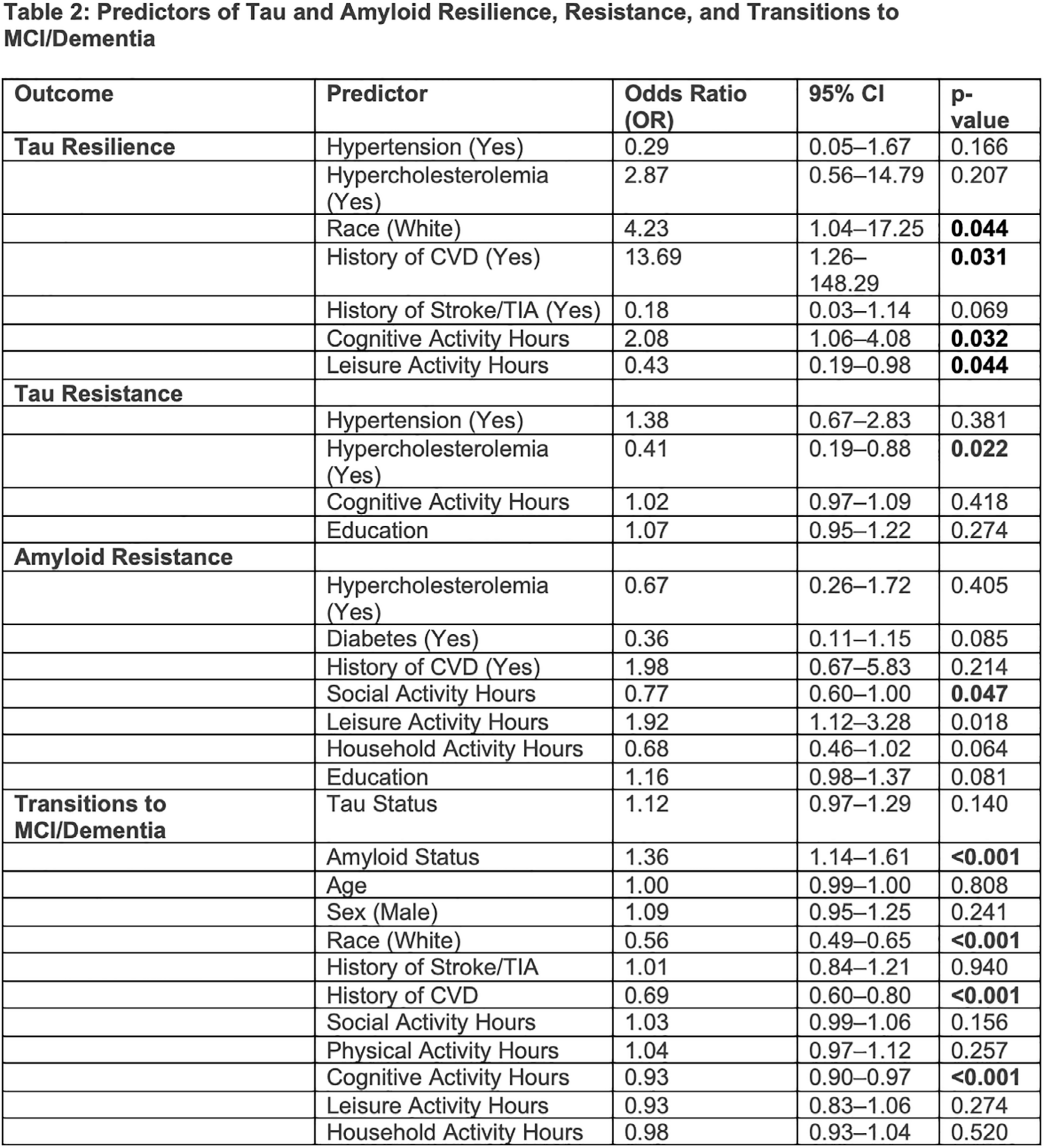# Longitudinal Trajectories of Cognitive Impairment: Predictors of Tau and Amyloid Resistance & Resilience

**DOI:** 10.1002/alz70856_097169

**Published:** 2025-12-24

**Authors:** Daniel Willie‐Permor, Oscar L Lopez, Steven E. Reis, Anum Saeed, Beth E. Snitz, Brian J Lopresti, Victor L Villemagne, M. Ilyas Kamboh, Neelesh Nadkarni, C. Elizabeth Shaaban, Ann D Cohen

**Affiliations:** ^1^ University of Pittsburgh, Pittsburgh, PA, USA; ^2^ Alzheimer's Disease Research Center (ADRC), PITTSBURGH, PA, USA; ^3^ University of Pittsburgh Alzheimer's Disease Research Center (ADRC), Pittsburgh, PA, USA; ^4^ University of Pittsburgh School of Medicine, Pittsburgh, PA, USA; ^5^ University of Pittsburgh, School of Medicine, Pittsburgh, PA, USA; ^6^ Department of Human Genetics, University of Pittsburgh, Pittsburgh, PA, USA

## Abstract

**Background:**

Cognitive impairment (CI) in Alzheimer's disease (AD) is driven, among others in the AT(V)N framework, by tau and amyloid pathology, but individual responses to these pathologies vary widely. Some individuals maintain cognitive function despite significant pathology (resilience), while others remain pathology‐free and cognitively intact (resistance). Emerging evidence suggests that health behavior factors, such as physical activity, cognitive engagement, and social interaction, along with demographic and clinical variables, may play a protective role. Our aim was to identify predictors of resistance and resilience.

**Method:**

This study included 152 participants from the Heart Strategies Concentrating on Risk Evaluation (Heart SCORE) study with longitudinal imaging data and cognitive assessments. Tau burden was measured using [18F]Flortaucipir (FTP) and amyloid burden using [11C]Pittsburgh Compound‐B (PiB) PET imaging. Cognition was categorized clinically as unimpaired or impaired (MCI or dementia) cognition. Resistance and resilience profiles were developed by combining tau, amyloid, and CI status (e.g., tau‐resilient: tau‐positive, CI‐negative; tau‐resistant: tau‐negative, CI‐negative). Stepwise logistic regression with a *p*‐value cutoff of 0.2 was used to identify predictors of tau and amyloid resistance and resilience. Discrete‐time survival analysis (logistic regression) was used to examine transitions to MCI/dementia. Model performance was assessed using the Area Under the ROC Curve (AUC) for discrimination and calibration plots for the accuracy of predicted probabilities. Calibration plots were generated using local regression (loess) and evaluated for internal validity.

**Result:**

The demographic, clinical and health behavior characteristics of participants stratified by cognition status at baseline visit are summarized in Table 1. White race, history of CVD, cognitive activity were positively associated with tau resilience, while leisure activity was inversely related. Hypercholesterolemia showed an inverse association with tau resistance. Social activity, leisure activity, household activity, and education were positively associated with amyloid resistance. Within every year of study follow‐up, amyloid positivity and tau‐positivity predicted higher odds of progression to MCI/dementia, while CVD history, White race and cognitive activity predicted reduced lower odds. (See Table 2 & Figure 1 for model discrimination/calibration).

**Conclusion:**

Tau and amyloid status influence cognitive transitions, with health behavior factors, demographic and CVD history playing key roles in resistance and resilience.